# Measurement of student engagement in health professions education: a review of literature

**DOI:** 10.1186/s12909-023-04344-8

**Published:** 2023-05-20

**Authors:** Salah Eldin Kassab, Mohamed Al-Eraky, Walid El-Sayed, Hossam Hamdy, Henk Schmidt

**Affiliations:** 1 College of Medicine, Gulf Medical University, Ajman, United Arab Emirates; 2 Faculty of Medicine, Suez Canal University, Ismailia, Egypt; 3 College of Dentistry, Suez Canal University, Ismailia, Egypt; 4 Institute for Medical Education Research, Erasmus University Rotterdam, Rotterdam, Netherlands

**Keywords:** Student engagement, Students as partners, Measurement, Medical, Dentistry, Nursing, Pharmacy, Health sciences, Nutrition, Physiotherapy

## Abstract

Student engagement is a complex multidimensional construct that has attained great interest in health professions education (HPE). Definition and conceptualization of student engagement is an important step that should drive the development of the instruments for its measurement. We have recently proposed a comprehensive framework for student engagement in HPE with a definition of engagement as student investment of time and energy in academic and non-academic experiences that include learning, teaching, research, governance, and community activities. The dimensions of student engagement in this framework included the cognitive, affective, behavioral, agentic, and socio-cultural. Guided by the student engagement framework, this non-systematic review aims to identify, critically appraise, and summarize the existing methods for measuring student engagement in HPE. Extrapolating from higher education literature, we attempted to link the theoretical perspectives of student engagement with the published methods of its measurement in HPE context. In addition, we have described the different methods of measuring student engagement including self-report surveys, real time measures, direct observation, interviews/focus groups, and the use of multiple instruments. The span of engagement dimensions measured by self-report surveys ranges from one to five dimensions. However, measurement of agentic and sociocultural dimensions of engagement in HPE is still limited and further research is required. We have also reflected on the existing methods of measuring engagement of students as active partners in HPE. The review also describes the advantages, limitations, and psychometric properties of each method for measuring student engagement. We ended the review with a guiding conclusion on how to develop and select an instrument for measuring student engagement in HPE. Finally, we addressed the gaps in the literature about measuring engagement of HPE students and future research plans.

## Background

The research agenda on student engagement in education has witnessed a progressive rise during the past three decades. The main drive for this rise is the significance of student engagement as a predictor of academic success, well-being, satisfaction, increased retention, decreased burnout, and enhanced self-directed learning [1]. Furthermore, engagement of students in learning enhances teacher motivation [2]. Accordingly, engagement of students has been used as an indicator for the quality of medical programs [3] and a measure of institutional excellence in medical education [4]. We have also recently reviewed the different aspects related to this important construct and its implications on health professions education [1, 5]. Despite the presence of several instruments in the literature for measuring engagement of HPE students, there are no currently existing comprehensive reviews that describe these methods. There are also no guiding principles on how to develop and select an instrument for measuring student engagement in HPE.

### Definition of student engagement

Previous literature in higher education has included several definitions of student engagement according to the underlying theoretical perspectives. The prevailing three theoretical underpinnings that explain student engagement include the psychological, behavioral, and psychosocial perspectives [1, 6]. The psychological perspective considers engagement as an internal psychological state of students. According to this perspective, student engagement is defined as the students’ psychological state of activity that makes them feel activated, exert effort, and be absorbed during learning activities and students’ state of connection with the school community [7]. The behavioral perspective explains engagement as both the student behavior and the institutional factors that drive the student engagement. Accordingly, student engagement from this perspective is defined as the time and effort students dedicate to educationally purposeful activities and the practices that institutions apply to motivate students to participate in these activities [8]. The sociocultural perspective addresses the role of social, cultural, and political factors in student engagement. Accordingly, sociocultural engagement is defined as the student ability of expanding viewpoints and providing awareness of, and appreciation for, others from diverse social and cultural backgrounds [9].

### Student engagement is multidimensional and multi-level

The multidimensional nature of student engagement as a construct poses a practical difficulty to its measurement. Student engagement is conceptualized into behavioral, cognitive, emotional, agentic, and sociocultural dimensions that are measured by relevant indicators. Behavioral engagement can be measured by indicators such as student attendance, participation in curricular or extracurricular activities, effort, and ability to persevere in academic pursuits despite challenges. Indicators of emotional engagement include the emotions students experience towards their learning, peers, faculty, and school. These emotions include happiness, enthusiasm, pride, enjoyment, and feeling of bonding. Cognitive engagement includes absorption in learning, metacognition, perceived value of academic tasks and use of high-order cognitive skills. Agentic engagement indicates the student power to influence to their education, their future lives, and their social environment [10]. Indicators of agentic engagement inside the classroom include the active contribution of students to their learning process [11]. Agentic engagement outside the classroom can be measured by the student involvement in teaching of their peers, active participation in school governance, and involvement in community activities. Sociocultural engagement refers to the extent of students’ awareness of, and appreciation for, the diverse perspectives and experiences represented in their learning community. Indicators of sociocultural engagement include appreciation for different cultural backgrounds, willingness to engage in cross-cultural dialogue, and accepting to learn from others from different perspectives [9].

Student engagement can be molded according to the changes in the surrounding environment. These changes will have direct implications on the methods used for its measurement. For example, student engagement varies according to the type of learning activity (e.g., large classroom, small group learning, self-learning). Similarly, engagement of students differs according to the time scale, which can range from an engagement in a short learning activity to engagement along the duration of a course or a program.

### Spheres of student engagement

The spheres of students’ engagement include either engagement in their own learning or engagement as partners in education. The areas of engagement as partners include provision of education, scholarly research, governance, and community activities [4]. Accordingly, student engagement has been defined as academic experiences of students in learning, teaching, and research, at the cognitive, behavioral, and emotional levels through interactions with peers, faculty, and college community [12]. We have recently provided a comprehensive definition of student engagement in HPE as the student investment of time and energy in academic and non-academic experiences that include learning, teaching, research, governance, and community activities. Students are involved in these aspects at the cognitive, affective, behavioral, agentic, and socio-cultural dimensions [1].

### Student engagement in technology-enhanced learning (TEL) environments

Technology-enhanced learning (TEL) environments are essentially conducted online and offer both opportunities and challenges for student engagement. These environments offer more flexibility and autonomy for students to customize their learning experience according to the most suitable time, method, and place. However, TEL requires students to possess the technology literacy that allows them to navigate online platforms, manage multimedia resources, and manage digital information. Furthermore, students require adaptation to different methods of communication and social skills compared with face-to-face settings to get engaged in online learning activities. Therefore, methods of measuring student engagement in face-to-face learning environments may not be suitable for use in online environments. For example, direct observation is suitable for measuring behavioral engagement of students through direct interactions in face-to-face classrooms. However, measuring behavioral engagement of students in an online environment may need to rely on other measures such as self-report surveys, data analytics and log files.

This review aims to provide an overview of the methods of measuring student engagement in HPE. We linked each method with the underpinning theoretical perspectives, and described the measured engagement dimensions, advantages and limitations, and the psychometric properties of each method. In addition, we addressed the current gaps in the literature about measuring student engagement in HPE and directions for future research.

### Methodology

This manuscript represents a non-systematic literature review employing an explicit search strategy to reduce the biases in selection of included articles. The review included articles published in English with the focus of research on conceptualization and measurement of student engagement, and the main subjects are HPE students. We conducted literature search using the following databases: MEDLINE, PubMed, ProQuest, SCOPUS, Education Resources Information Centre (ERIC), Science Direct, and EBESCO. We searched for peer-reviewed research articles in the databases by title and abstract using key terms such as student engagement, engagement, learner engagement, students as partners, and partnership. We combined the previous terms with other words such as health professions, medical, dentistry, pharmacy, nursing, allied health professions, clinical psychology, physical therapy, nutrition, and occupational therapy. In addition, we selected relevant articles from the references list of identified key publications in student engagement. The literature search was limited to articles between 1990 and November 2022. Searching the databases yielded 3019 articles, while an additional 617 articles were identified through searching the listed references and hand-searching of HPE journals. Following deduplication and excluding irrelevant articles through screening of titles and abstracts, 144 articles were selected. Individual screening of full text articles by the two authors (SK and WE) led to the selection of 71 articles to be included in the review with a date of publication ranging from 2003 to 2022. Although the main emphasis of the review is on studies relevant to HPE context, authors agreed to include additional relevant articles (*n *= 19) related to non-HPE contexts. Articles that used self-reports with only one statement for measuring student engagement were excluded from the review. We used EndNote (version 7, Clarivate Analytics, Philadelphia, United States) as the reference manager software for the bibliography of the identified articles.

### Methods of measuring student engagement in HPE

**Fig. 1:**
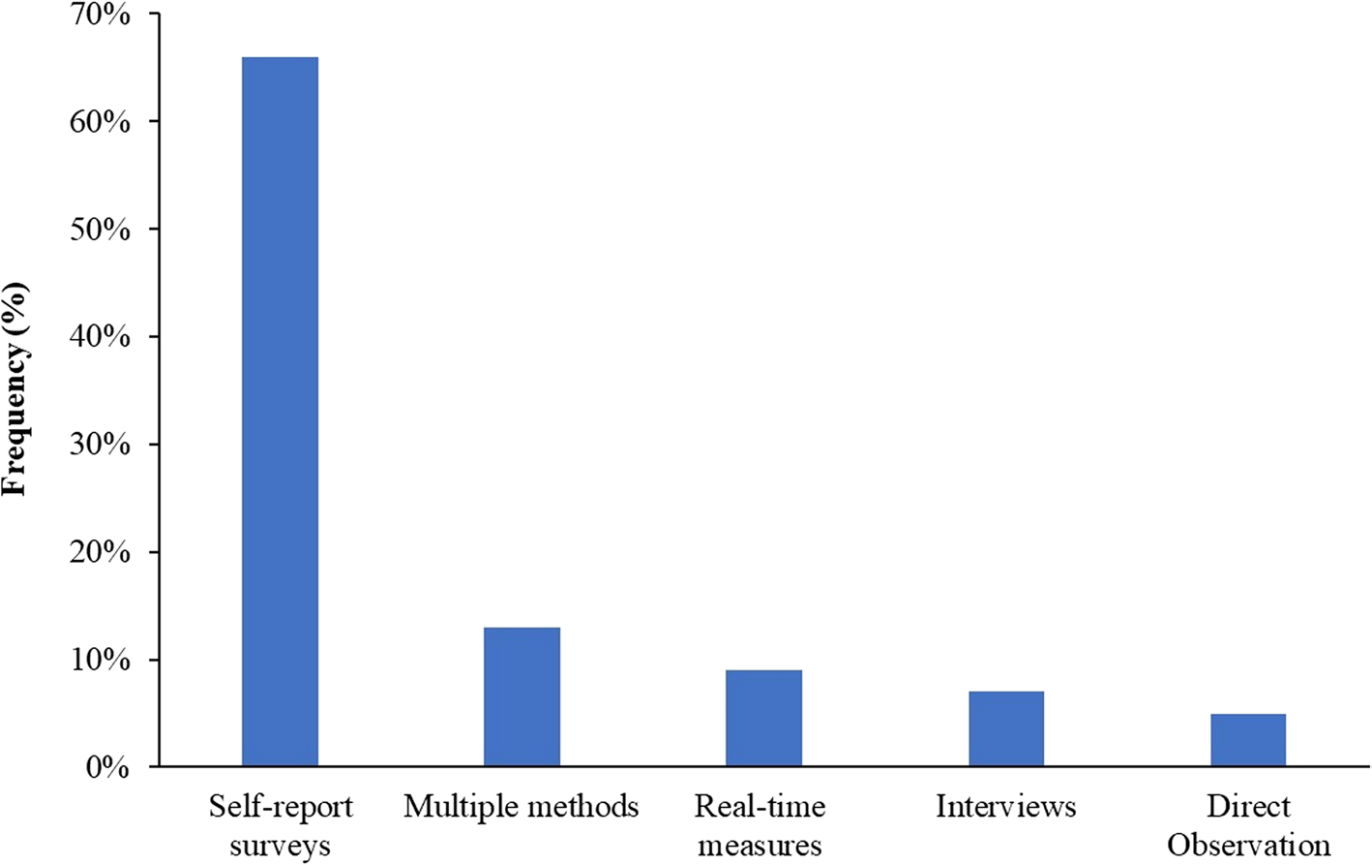
Frequency of the methods for measuring student engagement in health professions education

The outcome of the literature search resulted in different measures of student engagement in HPE. The common methods of measuring student engagement are self-report surveys, real-time measures, direct observation, interviews, or a combination of more than one method (Fig. 1).

- **Self-report surveys**

Self-report surveys are the most used methods for measuring student engagement in HPE, representing approximately two thirds of the reported methods. These surveys are easy to administer, cheap, and can sample many students in a brief period. Furthermore, self-reports can measure unobservable aspects of engagement such as cognitive and emotional dimensions [13]. The number of dimensions measured by the self-reports varies from one to five dimensions. However, the problem with self-reports is the inability to measure the dynamic nature of student engagement in certain learning situations. One attempt to overcome this shortcoming is the experience sampling using short questionnaires that are distributed several times during a learning activity [14]. Most surveys are not able to capture the complex nature of the student engagement construct. Even some of the questionnaires that cover the multiple dimensions of engagement lack the alignment between conceptualization and the method of measurement. Below is a detailed description of the common self-report measures in HPE literature and a summary of these instruments in provided in Table 1.

**Table 1:** Self-report surveys used for measuring student engagement in health professions education

Name of the instrument	Underlying theoretical perspective-model	Dimension(s) of engagement	Number of items	Grain size of measurement	Measurement setting	References
**Self-reports measuring one dimension**						
Situational cognitive engagement questionnaire	Psychological-Flow theory ^a^	Cognitive	4 items	Learning activity	Face-to-face (PBL, TBL)	[14, 15]
Learners’ Engagement and Motivation Questionnaire	Psychological-Flow theory	Cognitive	6 items	Learning activity	Multimedia learning	[16]
**Self-reports measuring two dimensions**						
User Engagement Scale-20 (UES-20)	Psychological-Flow theory	▪ Cognitive ▪ Emotional	20 items	Learning activity	e-learning resources	[17]
User Experience Questionnaire (UX questionnaire)	Psychological-Flow theory	▪ Cognitive ▪ Emotional	8 items	Learning activity	Immersive virtual learning	[18]
**Self-reports measuring three dimensions**						
Utrecht Work Engagement Scale (UWES)	Psychological-Schoolwork engagement model	▪ Cognitive ▪ Emotional ▪ Behavioral	3, 9, 14, & 15 items	▪ Course ▪ Program	Face-to-face	[19–29]
University Student Engagement Inventory (USEI)	Psychological-Engagement as a meta-construct	▪ Cognitive ▪ Emotional ▪ Behavioral	15 items	▪ Program ▪ University	Face-to-face	[30–32]
Classroom Engagement Survey (CES)	Psychological-Engagement as a meta-construct	▪ Behavioral ▪ Emotional ▪ Cognitive	8 items	Learning activity	Face-to-face—classroom	[33–35]
TEL engagement scale	Psychological-Engagement as a meta-construct	▪ Satisfaction (Emotional) ▪ Goal setting & planning (Cognitive) ▪ Physical interaction (behavioral)	19 items	Learning activity	Online learning (TEL resources)	[36]
**Self-reports measuring four dimensions**						
Online Student Engagement Scale (OSE)	Psychological-Engagement as a meta-construct	▪ Skills (Cognitive) ▪ Emotional ▪ Participation (Behavioral) ▪ Performance	19 items	Course	Online learning	[37–40]
College students’ learning engagement scale in cyberspace	Psychological-Engagement as a meta-construct	▪ Cognitive ▪ Emotional ▪ Behavioral ▪ Interactive	19 items	Course	Online learning	[41]
**Self-reports measuring student engagement at the institutional level**						
National Survey for Student Engagement	Behavioral perspective	▪ Cognitive ▪ Behavioral ▪ Agentic ▪ Sociocultural	≈ 80 items ^b^	Program	Face-to-face	[42–47]
**Self-reports measuring student engagement as partners**						
Educational student engagement scale	Sociocultural perspective – Positioning theory & CoP^c^	Agentic	6 items	Program	Face-to-face	[48]

^a^ Flow theory is applied to one of the four items only

^b^ Institutions may also add optional or customizable items to tailor the survey to their specific needs

^c^ CoP = Community of Practice

1. ***Self-reports measuring one engagement dimension***

### Situational cognitive engagement questionnaire

This questionnaire measures cognitive engagement defined as a psychological state in which students exert a significant amount of effort to understand the topic at hand and in which they persist studying over a prolonged period [15]. Items are scored on a 5-point Likert scale, ranging from 1 (not true at all) to 5 (very true for me). The validity of the questionnaire has been tested in a large sample of applied science students with model fit statistics supporting the model [15]. Furthermore, coefficient H was 0.93 and 0.88 when evaluated on students in applied sciences and medicine [14, 15], respectively. Because of the short nature of this questionnaire, it allows multiple measurements of cognitive engagement in response to contextual changes. However, the limited scope of this questionnaire to the cognitive dimension does not allow measurement of other relevant engagement dimensions during collaborative learning in PBL tutorials such as emotional, behavioral, and social dimensions. Another limitation of the questionnaire is the use of behavioral engagement indicators such as effort and persistence as measures of cognitive engagement.

### Learners' engagement and motivation questionnaire

This questionnaire has been used for measuring cognitive engagement of health professions education students in multimedia learning [16]. The questionnaire is based on conceptualizing student engagement as a state of flow (absorption, full concentration, intense enjoyment, and distortion of time awareness). The instrument consists of six items measured on a 7-point Likert scale where 1= not at all true of me to 7=very true of me. Cognitive engagement is conceptualized into three subconstructs: 1) Attention focus (2 items), 2) Intrinsic interest (2 items), and 3) Curiosity (2 items). Although the instrument is declared for measuring cognitive engagement, indicators were a mix of behavioral (attention) and emotional (interest and curiosity) engagement. However, the scale has been pre-validated for internal structure and exploratory factor analysis demonstrated a unidimensional factor structure. Internal consistency reliability using Cronbach alpha was 0.93 to 0.95.

2. ***Self-reports measuring two engagement dimensions***

### Classroom engagement survey (CES)

The CES was initially designed in general education settings and imported for use in nursing education [33]. The CES consists of 8 items designed to measure behavioral and emotional engagement of students [33]. Behavioral engagement is measured by student participation in the classroom (five items) and emotional engagement measures their enjoyment (three items). Another version of the CES consists of 8 items representing the behavioral (3 items), emotional (3 items), and cognitive (2 items) dimensions [34, 35]. However, a major limitation of this version is the lack of validity support for the multidimensionality of the construct. In addition, two items are measuring behavioral and cognitive engagement at the group level rather than individual students. Items are scored on a five-point Likert scale (1, strongly disagree; 2, disagree; 3, neither agree nor disagree; 4, agree; 5, strongly agree). The summed scores range from 5 to 40. A higher score indicates greater engagement of students with a score of 24 considered as a neutral score. The use of CES in HPE demonstrated an internal consistency reliability of the questionnaire ranged from Cronbach’s alpha coefficient range from 0.83 [34] to 0.88 [33, 35].

### User Experience Questionnaire (UEQ)

The long version of the UEQ (68 items) has been developed and validated for measuring the experience in immersive virtual environments using participants mainly from information and communication technology or computer sciences [49]. The shorter version of the questionnaire has been adapted for use for measuring engagement of medical students during the use of 360^O^ videos in Anatomy education [18]. This version consists of 8 items that measure student engagement as a state of flow characterized by immersion (2 items), enjoyment (2 items), loss of time awareness (2 items), and overall involvement (2 items). Students are asked to score their degree of agreement with each statement on a scale of 0–100, and the average score represents the degree of student engagement. The main limitation of the UEQ is the lack of evidence for the construct validity either for its internal structure using factor analysis or criterion-related evidence by testing relationships to other variables.

### User Engagement Scale-20 (UES-20)

The UES-20 has been developed for measuring engagement of commercial users with the learning resources to which they are exposed in online environments [50]. The scale was then imported for measuring engagement of medical students during their e-learning for diagnostic imaging using adaptive tutorials [17]. Adaptive tutorials are intelligent online tutoring systems, which provide a personalized learning experience of students through immediate feedback that is modified according to individual student responses [17]. The instrument measures the cognitive and emotional dimensions of engagement with e-learning resources. The scale consists of 20 items scored on a 5-point Likert scale (1 = strongly disagree and 5 = strongly agree) clustered under four subscales. The subscales include focused attention (ability to concentrate and absorb information), perceived usability (affective and cognitive responses to the resource), novelty and involvement (level of triggered interest, feeling of immersion and having fun), and aesthetic appeal (impression made by the visual appearance of the user interface). Factor analysis demonstrated a four-factor structure, which supports the multidimensionality of the questionnaire [50] [17].

3. ***Self-reports measuring three engagement dimensions***

### Utrecht Work Engagement Scale (UWES)

This scale is one of the most used self-reports in HPE and has an established theoretical basis on the psychological perspective of engagement and the schoolwork engagement model. According to this model, engagement is conceptualized as a positive state of study-related fulfillment characterized by vigor (emotional), dedication (cognitive), and absorption (behavioral) [51]. Different versions of the scale have been used in the HPE literature with larger versions consisting of 17 items [19, 20], 15 items [21, 22], and 14 items [23–25]. Shorter versions that consist of 9 items [26–28] and even 3 items [29] are also used. For each of the items, students are asked to identify their level of engagement using a seven-point Likert scale (1 = never to 7 = always). The sum scores of items are divided by the number of items in the scale to represent total engagement score. The different versions of the scale have demonstrated good psychometric properties in various contexts as evidenced by good to excellent internal consistency reliability and factor analysis findings that support the theoretical model [19, 23, 25]. Other sources of validity evidence for the questionnaire are the negative correlations between student engagement scores using UWES and perceived stress [21, 25] and burnout [21, 24, 28]. In addition, there is significant positive association between perceived satisfaction of basic psychological needs (autonomy, competence, and relatedness) and student engagement [28]. Furthermore, student engagement is promoted by students’ academic self-efficacy beliefs, students’ active self-care, and resilience [27].

### University Student Engagement Inventory (USEI)

The earlier version of the USEI was originally developed in Portuguese to measure student engagement [52]. This version is composed of 32 items distributed in three dimensions covering behavioral (11 items), emotional (10 items), and cognitive (11 items) engagement. However, a reduced version that consists of 15 items has demonstrated better psychometric properties [30]. The short version has been used for measuring engagement of medical [31], dental [30], and pharmacy [32] students. Questionnaire items are distributed in a three-factor structure covering the three dimensions of engagement: behavioral (5 items), emotional (5 items), and cognitive (5 items). Items are assessed on a 5-point scale ranging from 1 (never) to 5 (always). An evidence of construct validity of the scores from the reduced version has been demonstrated by confirmatory factor analysis, which supported the three-factor model with significant correlations between the subscales [30, 31]. In addition, emotional and behavioral engagement scores correlated negatively with perceived burnout [32]. The questionnaire has also demonstrated an acceptable reliability (≥ 0.7) using composite reliability (CR) and Cronbach’s alpha [30].

### Technology-enhanced Learning (TEL) engagement scale

This scale intends to measure the engagement of students in TEL resources, with an example of its application in Anatomy education [36]. The instrument consists of 19 items scored on a five-point Likert scale (1 = strongly disagree and 5 = strongly agree). Exploratory factor analysis yielded a three-factor structure as follows: satisfaction (8 items), goal setting and planning (7 items), and physical interaction (4 items). These emerging factors generally conform with emotional, cognitive, and behavioral dimensions of engagement, respectively. In addition, there are significant correlations between the three engagement dimensions. TEL engagement score is calculated by summing the responses from each item with the minimum score of 19 and a maximum score of 95. The reported internal consistency reliability of the scale was 0.86 with acceptable reliability of each of the three engagement dimensions. Although the scale has proved evidence of validity in Anatomy learning resources, the outcome of its application in other HPE subjects is unclear.

4. ***Self-reports measuring four engagement dimensions***

### Online Student Engagement Scale (OSE)

This scale attempts to measure the behavior, thoughts, and feelings of students in online learning. The initial version of the scale has been adapted from the Student Course Engagement Questionnaire (SCEQ) for use in online communication engineering courses [37]. The questionnaire was then imported for use in nursing education [37–39]. It comprised 19 items divided into four factors: 1) skills engagement (cognitive), 2) emotional engagement, 3) participation engagement (behavioral), and 4) performance engagement [38, 40]. The items are scored on a 5-point Likert scale using the following response categories: very characteristic of me (5), characteristic of me (4), moderately characteristic of me (3), not really characteristic of me (2) or not at all characteristic of me (1). The total engagement score represents the sum for the four engagement dimensions with ninety-five considered as the maximum score. The scale demonstrates high internal consistency reliability with a Cronbach alpha ranging from 0.91 [37, 38] to 0.95 [39]. In addition, factor analysis yielded a four-factor structure including skills, emotional, participation, and performance [37]. The main disadvantage of this questionnaire is mixing between the dimensions and outcomes of engagement (performance engagement).

### College students’ learning engagement scale in cyberspace

This scale has been used for measuring learning engagement in online courses for Chinese nursing students [41]. Authors conceptualized online engagement into four dimensions: behavioral, emotional, cognitive, and interactive engagement. The scale consists of 19 items rated on a 5-points Likert scale ranging from 1 (completely inconsistent) to 5 (completely consistent), with a total score of 19 to 95. A higher total score indicated higher online learning engagement. Confirmatory Factor Analysis (CFA) showed that the structure of this scale was stable and the Cronbach's Alpha coefficient was 0.972.

5. ***Self-reports measuring student engagement as partners***

### Educational student engagement scale

This questionnaire measures the engagement of medical students as active partners in the provision of education [48]. The questionnaire consists of six items designed to be in line with the ASPIRE criteria for institutional excellence in student engagement [4, 53]. The items cover mainly the agentic engagement dimension and include student role in curriculum evaluation, peer teaching, self- and peer assessment, and the role of their feedback on curriculum development. Students are asked to indicate their level of agreement on each item based on a five-point Likert scale where 1= strongly disagree and 5 = strongly agree. The average score of the six items represents the mean level of student engagement. The internal consistency reliability of the questionnaire is 0.88. The questionnaire has also demonstrated evidence of predictive validity by the positive relationships between student engagement scores and student learning outcomes. However, the scope of the questionnaire is limited to measuring engagement in provision of education and does not measure the other aspects of student engagement as partners such as engagement in governance, scholarly research, and community activities.

6. ***Measures of institutional level of student engagement***

### National Survey of Student Engagement (NSSE)

The NSSE originated in the early 20^th^ century as a tool for measuring student engagement at the institutional level to improve the undergraduate experiences of students, document effective institutional practices, and benchmark between higher education institutions [8]. This self-report survey measures student engagement on an annual basis and is designed based on the behavioral perspective of student engagement. The instrument consists of approximately 80 items with 10 indicators representing five themes of student engagement in addition to 6 high impact practices. The themes include academic challenge (4 indicators), learning with peers (2 indicators), experiences with faculty (2 indicators), and campus environment (2 indicators). The academic challenge theme covers higher-order learning, reflective & integrative learning, learning strategies, and quantitative reasoning. Learning with peers includes collaborative learning and discussions with diverse others. Experience with faculty includes student-faculty interaction and effective teaching practices. Campus environment includes quality of interactions and supportive environment. High impact practices include service-learning, learning community, research with faculty, internship or field experience, study abroad, and culminating senior experience. In the HPE research context, NSSE has been used for measuring engagement of nursing students [42, 43]. The main limitation of the NSSE is mixing between indicators, drivers, and outcomes of engagement. One of the surveys developed by items borrowed from NSSE is the Survey of Student Engagement (SSE) instrument. The SSE instrument has been validated [44] and used for measuring engagement of medical students [45–47]. The instrument consists of 14 items grouped under three categories: 1) collaborative learning (4 items), 2) cognitive development (five items), and 3) personal skills (4 items). Items are scored on a 4-point Likert scale ranging from 1 = Never to 4 = very often, and the total engagement score is the sum of the scores in the three dimensions.

### Classroom Survey of Student Engagement (CLASSE)

CLASSE is an adapted version of NSSE to measure engagement of students at the classroom level [54]. The purpose is to provide feedback to institutions on how to enhance instructional practices for better engagement of students. The CLASSE questionnaire consists of 39 items [55] with a shorter version of 19 items [56]. Each is assessed on a 4-point Likert scale (0 = never, 1= one time, 2= three to five times, and 3= more than five times) and then multiplied by 33.3 to produce a score ranging from 0 (lowest) to 100 (highest). The questionnaire consists of three dimensions: 1) active and collaborative learning, 2) student –faculty interaction, and 3) level of academic challenge. The questionnaire demonstrated good internal consistency reliability in HPE settings with a Cronbach’s alpha = 0.89 for the 39-item version [55].

b) **Direct observation**

Direct observation can measure the engagement of individual students or the whole classroom during a learning activity. The observable indicators for student engagement are mainly focused on the behavioral dimension such as attention, asking questions, and participating in classroom activities. Observation measures can provide real-time changes in student engagement and describe rich information about the contextual factors affecting engagement. However, they are time-consuming, require training of observers, and limited to measuring the behavioral dimension of engagement. In addition, direct observation mostly includes a small number of students, which limits the generalizability of these measures. In the HPE literature, there are two reported direct observation methods, which are STROBE and In-class engagement measure (IEM).

1. **STROBE**

The name of the STROBE instrument refers to the strobe light that intermittently captures events at regular intervals. The instrument consists of 5-minute observational cycles repeated during the classroom activities. During each cycle, the trained observer records the behavior of the teacher and four students, and the process is repeated. Categories of student behaviors on the STROBE include: 1) Learner-to-learner engagement, such as speaking, listening, or both, 2) Learner-to-instructor engagement, such as speaking, listening, or both, and 3) Self-engagement: Learner reading, writing, or otherwise not visibly interacting with other learners or instructor. The observers also write free comments at the end of each 5-minute observation cycle. The reliability of the instrument measured by inter-observer agreement of observers who simultaneously scored the engagement of students was good to excellent [57]. In addition, validity-evidence was confirmed by the findings that student-student and student-instructor engagement were greater in PBL compared with traditional lectures [57, 58], and that engagement scores using STROBE correlated with student self-report of engagement [57].

2) **In-class engagement measure (IEM)**

The instrument is a revised form of STROBE to determine the level of engagement of students and teachers in the classroom settings on direct observation. Each observation cycle includes recording the behaviors of an instructor and four randomly selected students as snapshots for 5-min cycles [59, 60]. Each 5-min cycle consists of four 20-sec observations of individual learners. The observer scores student behavior on a scale 1 to 5 where 1 to 2 is non-participating personal without any communication and 3 to 5 is gradually increasing levels of participation and communication with the instructor and peers. The instrument demonstrated content-related validity evidence by review from education experts and criterion-related evidence of validity by the significantly higher engagement scores in active learning compared with traditional classes. In addition, the reliability of the instrument was proved by excellent inter-observer agreement in scores.

c) **Real-time measures**

Student engagement is a dynamic construct that responds to changes in the educational context. Therefore, several real-time measures have been developed to measure moment-to-moment changes in student engagement as it unfolds especially at the level of an educational activity. Examples of real-time measures applied for measuring student engagement in HPE are log files, physiological measures, and eye tracking. Log files are computer-generated data files that record system-related information including the internet usage patterns and activities. Log files can capture several indicators of behavioral engagement of students in technology-enhanced learning environments [61–69]. For example, a computer-generated engagement metric used multiple indicators to measure engagement of medical students with virtual patients such as time on page, MCQ answer accuracy, use of a clinical reasoning tool, and scores of students' written summary statements based on the VP encounter [69]. In addition, log files and physiological measures can automatically capture indicators of cognitive and emotional engagement (Table 2).

**Table 2:** Examples of real-time measures of student engagement in technology-enhanced learning (TEL) environments in health professions education

**Behavioral engagement**
Percent of student attendance
Number of times each student accessed an online course
Number of topics visited in discussion board
Number of posts read
Number of replies to online posts
Number of threads created
Number of posts click, likes, shares, or comments
Number of questions asked in discussion groups
Number of times student logged into the course Web site
Number of courses each student reported watching
Number of times each student completed the assignment
**Cognitive engagement**
Student completion of online tasks
Time spent online
Time spent on questions and answers
Time spent on class activities
Time students spent on the study material
Quality of the asked questions
Quality of student narrative responses to other students’ posts
Eye tracking
Heart rate EEG
**Emotional engagement**
Skin conductance
ECG
Facial expressions

For example, heart rate changes are used for measuring cognitive engagement of medical students in different types of class activities [70]. Eye-tracking is another indicator of engagement with the assumption that fixating the eyes on text or images for longer period indicate that students are cognitively engaged with the subject [71]. For example, eye-tracking has been used for measuring engagement of medical students with moulage simulations [72]. Real-time measures have the advantage of collecting large amounts of information in a short period. The collected data from real-time measures are usually precise because they are not subject to human errors or bias. However, analyzing this large volume of data could be challenging. In addition, these measures could be expensive and difficult to use in real educational environments [73].

d) **Interviews and focus groups**

Interviews and focus groups have the advantage of collecting in-depth information about student engagement. The collected information is usually deep and rich as students have the chance to explain how their engagement unfolds in a learning environment. By discussing with HPE students, they can explain the contextual factors that trigger or inhibit their engagement [74–80]. Students can also explain the types and characteristics of their engagement [81] and how they get engaged in learning activities [79]. Despite these advantages, collecting and analyzing qualitative data from interviews and focus groups are time-consuming and require training of interviewers [13]. In addition, the small sample of students and interviewer biases could limit the generalizability of conclusions about student engagement.

- **e) Multiple methods**

The comprehensive nature of the engagement construct made it almost impossible to measure all its components using a single instrument. To address this problem, several studies have used mixed quantitative and qualitative methods to triangulate the evidence about student engagement [72, 82–87]. Studies have also used multiple quantitative measures to capture more dimensions of this complex construct [88]. Another purpose of using multiple methods is to identify the contextual factors that drive/inhibit student engagement as well as the outcomes of engagement. For example, a study used self-report surveys, real-time measures, and interviews for measuring engagement of medical students in Anatomy and Histology by using digital games [82]. In this study, measures were not only focused on indicators related to the engagement construct, but also on antecedents and outcomes of engagement [82]. Another study used direct observation, work sample analysis, teacher rating, and student self-report to investigate the role of virtual patient simulations (VPS) in fostering student engagement [83]. The methods used were a mix between measuring flow (a state of engagement) and antecedents of engagement such as motivation, interest, and relevance [83]. Another study used both log files and focus group discussion to examine how visual learning analytics tools such as learning dashboards can support medical students’ cognitive engagement in the classroom [85]. The log files were used for measuring cognitive engagement while the focus group discussion explored the perceptions of students about their cognitive engagement [85]. Furthermore, a study used Immersion Score Rating Instrument (ISRI), engagement self-report, eye-tracking, and stimulated recall interviews to explore how the moulage authenticity impacts on student engagement [72]. Although multiple methods can capture different aspects of engagement, the real challenge is how to reconcile the inconsistency in the findings from different methods and combine these findings to achieve a more comprehensive image of student engagement.

### Conclusions and future directions

The measurement of student engagement in HPE has been a challenge despite the progressive interest of studying the construct for more than two decades. We conclude this review by highlighting important points to consider before developing and selecting instruments for measuring student engagement in HPE as shown in Table 3.

**Table 3:** Questions to ask before developing and selecting an instrument for measuring student engagement in health professions education

1. What is the theoretical perspective underpinning this method? (Psychological, behavioral, socio-cultural)
2. What are the dimensions of engagement to be measured? (Cognitive, behavioral, emotional, agentic, socio-cultural)
3. What is the sphere of measuring engagement? (Learning engagement, engagement in partnerships)
4. What is the grain size of measuring engagement? (Activity, course, program, University, extracurricular)
5. What is the time scale for measuring engagement? (Moment-to-moment, daily, long-term)

*First*, conceptualizing and defining student engagement is an important step in its operationalization and developing the appropriate methods of measurement. Furthermore, aligning the methods of measuring engagement with the underlying theoretical perspectives would streamline the comparison of findings across student engagement studies. Most of the available methods focus on measuring the psychological perspective of student engagement, while the behavioral perspective is limited to measuring student engagement at the institutional level. However, methods of measuring engagement from the socio-cultural theoretical perspective in the HPE literature are still in infancy. This is particularly relevant to the sphere of student engagement as partners in HPE education where theories such as Community of Practice (CoP) and Positioning theory are applicable [1].

*Second*, an important consideration during the design or selection of an instrument for measuring student engagement is identifying the dimensions of engagement that can be measured. Several instruments are available in the HPE literature for measuring behavioral, cognitive, and emotional engagement of students. However, the instruments designed to measure the agentic and socio-cultural dimensions of student engagement in HPE are limited and should be the target for future research.

*Third*, a plethora of publications in HPE address the measurement of student engagement in the sphere of own learning while measuring engagement of students as partners has been limited. Furthermore, most of the existing instruments in HPE literature about measuring student engagement as partners are not focusing on the engagement construct. Instead, the instruments used in these studies are measuring the drivers [78, 80, 89] and outcomes [84, 90] of student engagement. While the existing literature has provided valuable insights into the topic of student engagement as partners, further research is required to explore its practical applications and potential impact in HPE settings.

*Fourth*, student engagement has been conceptualized as a multi-level construct with variable time scales. Therefore, the granularity of measured engagement (learning activity, course, school, university) should be clearly identified at the outset of the study [7]. This issue has important implications on the appropriate method for each engagement level. For example, student engagement in a short learning activity can better be measured by direct observation or real-time measures. On the other hand, student engagement at the macro-level of the program can be measured by self-report surveys or interviews.

*Finally*, it is important to note that disengagement is not at the opposite end of the engagement spectrum. Engagement and disengagement are conceptually considered as distinct constructs with different outcomes. Accordingly, engagement and disengagement dimensions are distinct and require different structures of the instruments used for their measurement. Specifically, there is a lack of research on the measurement of student disengagement in HPE, highlighting the need for more studies to explore the different aspects related to this construct.

## Data Availability

Not applicable.

## Abbreviations

CES: Classroom engagement survey
CLASSE: Classroom Survey of Student Engagement
CoP: Community of Practice
HPE: Health professions education
IEM: In-class engagement measure
NSSE: National Survey of Student Engagement
OSE: Online Student Engagement Scale
TEL: Technology-enhanced Learning
UEQ: User Experience Questionnaire
UES-20: User Engagement Scale-20
USEI: University Student Engagement Inventory
UWES: Utrecht Work Engagement Scale
